# Metabolic and Blood Pressure Effects of Consuming Two Kiwifruit Daily for 7 Weeks: A Randomised Controlled Trial

**DOI:** 10.3390/nu14132678

**Published:** 2022-06-28

**Authors:** John Monro, Alex Lubransky, Suman Mishra, Jillian Haszard, Bernard Venn

**Affiliations:** 1 New Zealand Institute for Plant and Food Research Ltd., Palmerston North 4442, New Zealand; suman.mishra@plantandfood.co.nz; 2 Department of Human Nutrition, University of Otago, Dunedin 9054, New Zealand; alex.lubransky@gmail.com (A.L.); jill.haszard@otago.ac.nz (J.H.); bernard.venn@otago.ac.nz (B.V.)

**Keywords:** kiwifruit, antioxidant, fructose, blood pressure, fruit intake, Asian

## Abstract

Background: Eating two kiwifruit before breakfast by equi-carbohydrate partial exchange of cereal has been associated with lower postprandial glucose and insulin, but it increases the intake of fruit sugar. We assessed the effects of kiwifruit ingestion at breakfast over 7 weeks on metabolic and physiologic factors. Method: Forty-three healthy Asian participants were randomised to ingest 500 mL of carbonated water (control) or 500 mL of carbonated water plus two kiwifruit (intervention), before breakfast. Three-day weighed diet records were taken before and at week 4 during the intervention. Overnight fasting blood samples were taken at baseline and week 7. Forty-two participants completed the study (n = 22 control, n = 20 intervention). Results: The kiwifruit group consumed more fructose, vitamin C, vitamin E, and carbohydrates as a percentage of energy compared with the control group (*p* < 0.01). There was no evidence of between-group changes in metabolic outcomes at the end of the intervention, with the following mean (95% confidence interval) differences in fasting blood samples: glucose 0.09 (−0.06, 0.24) mmol/L; insulin −1.6 (−3.5, 0.3) μU/mL; uric acid −13 (−30, 4) μmol/L; triglycerides −0.10 (−0.22, 0.03) mmol/L; and total cholesterol −0.05 (−0.24, 0.14) mmol/L. There was a −2.7 (−5.5, 0.0) mmHg difference in systolic blood pressure for the intervention group compared with the control group. Conclusion: Eating two kiwifruit as part of breakfast increased fruit consumption and intake of antioxidant nutrients without a change in fasting insulin. There was a difference in systolic blood pressure and no adverse fructose-associated increases in uric acid, triglycerides, or total cholesterol. This simple intervention may provide health benefits to other demographic groups.

## 1. Introduction

The consumption of fruit is recommended by health authorities around the world, with current evidence of its benefit coming from an umbrella review in which fruit consumption was associated with a reduced risk of cardiovascular disease and possible protection against colon cancer, depression, and pancreatic disease [1]. Fruit intake has also been inversely associated with perceived stress [2]. In addition, recently, including fruit in a meal by partial carbohydrate exchange of starchy cereal was shown to substantially reduce glycaemic response [3]. Fruit may therefore play a valuable role in managing the effects of dysregulated lipid and glucose metabolism, which are a feature of metabolic syndrome. However, there is ongoing debate regarding the possible harmful effects of sugars, particularly those of fructose (fruit sugar) [4,5]. Lipid and glucose homeostasis was affected when free fructose was provided to people in amounts equating to 25% of their dietary energy intake [6]. In particular, uric acid is generated as a consequence of hepatic fructose metabolism, and this compound has been implicated in several conditions related to metabolic syndrome, including hypertension and cardiovascular disease [7,8]. Regardless of the food source, fructose has been found to raise the postprandial concentration of circulating uric acid [9]. However, it has been suggested that fructose from fruit in a normal diet is not harmful, whereas fructose from high intakes of added sugars (sucrose and high-fructose corn syrup) causes a metabolic disturbance, primarily due to the large amounts of sugars consumed [6,10].

Potentially countering the adverse health effects associated with sugars is the observation that antioxidant carotenoids in fruits and vegetables are inversely associated with fasting serum insulin, suggesting that these plant compounds may be beneficial against insulin resistance and diabetes [11]. Kiwifruit is a good source of antioxidants, ranking favourably in their total antioxidant capacity compared with other commonly consumed fruits, including grapefruit, apples, and pears [12]. In an acute kiwifruit intervention study, postprandial glycaemic and insulinaemic responses were lessened when healthy Chinese participants ate two kiwifruit 30 min before a standard breakfast [13]. This may be an important finding given that fasting insulin has been found to be an independent risk factor for metabolic syndrome in Asian population groups [14,15]. Therefore, an improvement in immediate postprandial outcomes just from eating two kiwifruit before breakfast could signal a simple healthful strategy if carried out over a longer period. From previous dietary intervention studies, 3–8 weeks has been a sufficient time to improve glycaemic, lipidaemic, and blood pressure outcomes [16,17]. Asian participants are likely to be a good cohort to test for metabolic and blood pressure effects of a kiwifruit intervention given that fruit intake among Asian populations is relatively low [18] and that the risk of hypertension was inversely associated with fruit intake in a Japanese cohort [19].

Following our previous work in which a kiwifruit preload to breakfast yielded acute metabolic improvements, the aim of the present study was to test the efficacy of a pre-breakfast kiwifruit intervention over 7 weeks on the primary outcome of fasting insulin, with secondary outcomes of glucose, lipids, blood pressure, and uric acid, in Asian participants. 

## 2. Materials and Methods

This was a randomised controlled dietary intervention study in which Asian volunteers were randomised to incorporate either carbonated water (control group) or carbonated water plus two kiwifruit (intervention group) into their breakfast each day for 7 weeks. Ripe Zespri^®^ SunGold kiwifruit (*Actinidia chinensis* var. *chinensis*) were provided by Zespri International Limited, Tauranga, New Zealand. Zespri advised that the energy content of the kiwifruit was 238 kJ/100 g, comprising 13.1 g of carbohydrate, 1.0 g of protein, and 0.3 g of fat. Participants were people who self-identified as being of East Asian descent living or studying in Dunedin, New Zealand. Recruitment was centred around the University of Otago and adjacent workplaces by advertising flyers and word of mouth. Potential participants were assured that there was no obligation to join the study and that they could withdraw at any time without the need to explain why and without penalty. Inclusion criteria were male or female gender, age 18 to 65 years, and East Asian ethnicity. Exclusion criteria were having undergone gastrointestinal surgery; a fasting blood glucose concentration exceeding 6.0 mmol/L; a body mass index (BMI) exceeding 30 kg/m^2^; pregnancy; or an allergy to kiwifruit. Respondents were given an information sheet and asked to confirm that he or she met the inclusion and exclusion criteria. The University of Otago Human Ethics Committee (Health) approved the study, reference H17/048, dated 23 April 2017. This study has been registered with the Australian and New Zealand Clinical Trials Registry, ACTRN12617000713392.

**Figure 1:**
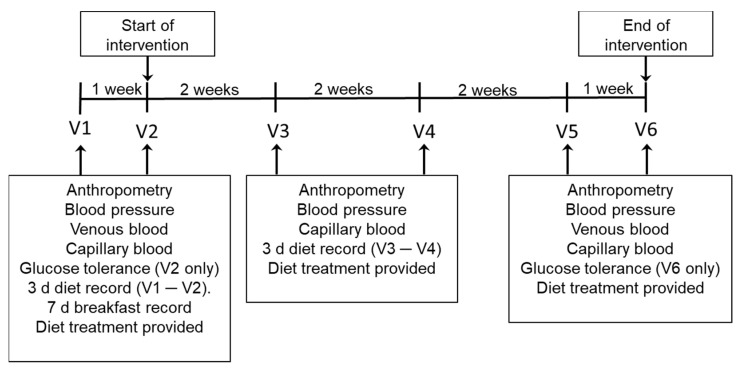
Timeline of the study showing the timing of blood sampling and dietary recording at each clinic visit (V).

A schematic of the study protocol over time is given in Figure 1.

### 2.1. Study Protocol

The primary outcome was fasting insulin concentration. Using a correlation of 0.7 between repeated fasting insulin measures (two measures at baseline and two final measures), a sample size of 36 (18 per group) was sufficient to detect a difference of 0.5 SD with 80% power at the 5% significance level. Forty-three people were recruited to allow for dropout. One week before the start of the intervention, participants attended the clinic for visit 1 (V1) to consent to take part in the study by signing a consent form and to receive instruction in dietary recording. During this lead-in week, participants recorded a breakfast log to allow the investigators and the participants subsequently assigned to the intervention group to discuss how to substitute kiwifruit for an equicaloric part of their regular breakfast. In addition to this breakfast log, all participants were provided with a set of electronic kitchen scales accurate to within one gram to use to fill in a three-day weighed diet record (Salter, Tonbridge, UK). Participants were randomised to the control group (500 mL carbonated water) or intervention group (500 mL carbonated water plus two gold kiwifruit) by the use of sealed envelopes and notified of their group at V2 (baseline). A randomisation sequence was created by the study statistician, who was otherwise uninvolved in the study administration, using Stata 16.1 (StataCorp, College Station, TX, USA), with a 1:1 allocation using random block sizes. Following baseline, participants were instructed to drink the carbonated water or to drink the water and eat the provided kiwifruit 30 min before they ate their main breakfast. The kiwifruit were fresh, ripe, export-grade fruit. Previous research in our laboratory [13], since confirmed [3], has established that kiwifruit consumed as a preload 30 min before breakfast by carbohydrate exchange substantially reduced the glycaemic response amplitude. Participants ticked a compliance check-sheet for each day that he or she ingested the allocated food or beverage. Participants attended the clinic every 2 weeks for clinical measures and to collect the study food. A second 3-day diet record was filled out during the fourth week of the intervention. The diet records were analysed for nutrient content using the New Zealand food composition database [20] embedded in FoodWorks 5 (Xyris Software, Brisbane, Australia).

### 2.2. Clinical Measures

Height was taken to the nearest millimetre using a stadiometer (Holtain, Crymych, UK). Weight was recorded to the nearest gram using a set of electronic scales (Seca Deutschland, Hamburg, Germany). A cloth tape measure was used to measure waist circumference to the nearest centimetre. Blood pressure was taken using an OMRON digital automatic blood pressure monitor, model HEM-907 (OMRON Corporation, Tokyo, Japan). Participants were seated with the arm to be tested resting on the arm of the chair such that the cuff of the monitor was at heart level. The monitor was positioned over the brachial artery, and three measurements were taken using the instrument’s average mode.

Overnight fasting blood samples were taken on four occasions from the anterior cubital fossa into one 10 mL Vacutainer™ tube (Becton, Dickinson and Company, Franklin Lakes, NJ, USA) and stored in a chilled insulated container. Within 2 h, whole blood was centrifuged at 2000 relative centrifugal force for 5 min at 4 °C, and plasma was aliquoted into 500 µL vials and stored in a −80 °C freezer until analysis.

Plasma uric acid concentration was determined by enzymatic colourimetric assay using a Roche uric acid reagent test kit. Plasma lipids were determined using Roche enzymatic colourimetric assays for total cholesterol and triglycerides on a Roche Hitachi Cobas c 311 auto-analyser (Roche Diagnostics, Indianapolis, IN, USA). Insulin concentration was determined using a Roche electrochemiluminescence immunoassay on a Cobas e 411 analyser according to the manufacturer’s instructions.

An oral glucose tolerance test was undertaken at baseline (V2) and the end of the intervention period (V6). Following an overnight fast, capillary blood glucose concentration was measured at baseline, after which participants ingested a 500 mL beverage containing 50 g glucose within 10 min. The blood glucose concentration was subsequently measured at 15, 30, 45, 60, 90, and 120 min after baseline. The incremental area under the curve was calculated using the trapezoidal method [21]. The capillary blood glucose concentration was measured using a HemoCue^®^ analyser (Hemocue Holding AB, Angelholm, Sweden). 

### 2.3. Statistical Analysis

All analyses were undertaken in Stata 16.1 (StataCorp, College Station, TX, USA). To determine the effect of the intervention on the main outcomes that had two baseline measures (at V1 and V2) and two final measures (at V5 and V6), mixed-effects regression models were used, with the final measures as the dependent variables, treatment as the independent variable, baseline measures as a covariate, and the participant as a random effect. The use of mixed-effects regression here takes advantage of the within-person variation from the repeated measures at each timepoint to increase efficiency. To describe how outcomes changed between baseline and the final measure for each group, means and standard deviations for the change, as well as for baseline measures, were reported. For the oral glucose tolerance test, which had only a single baseline and final measure, a linear regression model was used with the final measure as the dependent variable, treatments as the independent variables, and baseline measure as a covariate. Mean differences, 95% confidence intervals, and *p*-values were calculated. 

To describe the nutrient intake differences between the groups, linear regression models were used with the intake from V4 as the dependent variable, treatment as the independent variable, and intake from V2 as a covariate. Residuals of all regression models were plotted and visually assessed for homogeneity of variance and normality. 

## 3. Results

**Figure 2:**
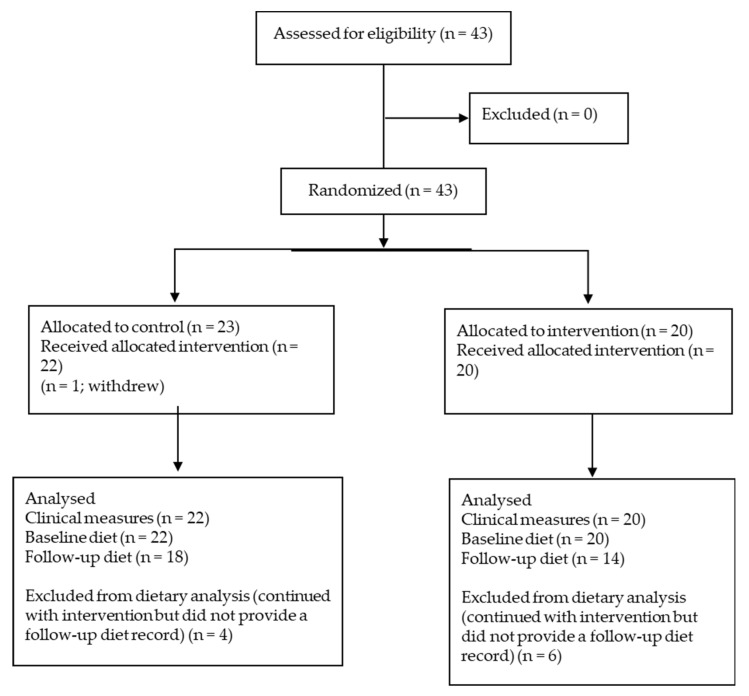
Flow of participants.

The control group had 95% compliance in drinking the carbonated water. Compliance for the intervention group was 87% for the carbonated water and 92% for the kiwifruit. The flow of participants is given in Figure 2.

The demographic and baseline characteristics of the 43 participants are given in Table 1. The age of participants ranged from 19 to 36 y. Most participants were of Chinese ethnicity (70% in the control group and 80% in the intervention group), with all other participants identifying as either Malay (n = 5), Taiwanese (n = 3), Korean (n = 2), Japanese (n = 2), Filipino (n = 1), Vietnamese (n = 1), South-East Asian (n = 1), or New Zealand Asian (n = 1). Ten people (4 in the control group and 6 in the intervention group) did not provide a second diet record.

Mean ± SD vegetable intake was lower in the kiwifruit group (120 ± 129 g/d) compared with the control group (242 ± 202 g/d) at baseline, with no evidence of a difference between groups at 7 weeks (203 ± 270 g/d and 240 ± 378 g/d, respectively). At baseline, the control and intervention groups had mean (SD) fruit intakes of 0.7 (0.6) and 1.0 (1.3) portions per day, respectively. During the intervention, the mean fruit intake of the control group was 0.5 (0.8) portions per day, whilst it rose in the intervention group to 2.4 (0.8) portions per day. The mean (SD) daily energy intakes at baseline for the control and kiwifruit intervention groups were 8054 kJ/d (2872) and 8026 (1563) kJ/d, respectively. These energy intakes tended to reduce during the intervention by 797 kJ/d (95% CI: −2236, 643) and 55 kJ/d (−1045, 935) in the control and intervention groups, respectively, with no statistically significant between-group difference (*p* = 0.453). 

The nutrient intakes at breakfast, including the intervention foods, are given in Table 2. In both groups, there was a tendency for reduced energy intake at breakfast at 4 weeks compared with baseline, although the change was not significantly different between groups. Carbohydrates displaced fat as a percentage of energy in the intervention group, and there were increases in fructose and vitamins C and E in the kiwifruit group compared with the control group. 

The metabolic and clinical outcomes are given in Table 3. There were no statistically different changes in fasting insulin from baseline to 7 weeks in either group or when comparing the change between groups at 7 weeks. Similarly, there was no evidence of a difference in change over time between groups for the other metabolic outcomes or anthropometric measures. There was a significant decrease in systolic blood pressure for the intervention group compared with the control group.

## 4. Discussion

A main finding of the study was a greater intake of vitamins C and E and a shift in the macronutrient profile at breakfast, with carbohydrates contributing more and fat contributing less proportional energy in the kiwifruit group compared with the control group. There was no statistically significant difference in the primary outcome of fasting insulin between groups. However, there was a significant effect of the intervention on lowering systolic blood pressure by 2.7 mmHg over the study period, favouring the intervention group. 

The fasting insulin results are intriguing, as there was a non-statistically significant difference of 1.62 μU/mL between groups, with the greatest change over time occurring as an increase of 2.1 μU/mL in the control group. Differences of this magnitude are clinically meaningful, as they are associated with the risk of metabolic syndrome [15]. The lack of statistical significance in our participants may be due to the study being underpowered to detect a difference in fasting insulin or being of a shorter duration than required. It is also possible that more kiwifruit would create a larger difference, although the practicality of people eating more than two kiwifruit has not been tested. From other work, it appears to be difficult to lower fasting insulin using fruits and vegetables. In an intervention in which participants were encouraged to eat more fruit and vegetables, fasting insulin did not change over a 12-week period, even for participants consuming up to 5.5 extra portions of fruits and vegetables per day [22]. The authors speculated that the types of fruit and vegetables might influence the outcome, and it should be noted that one of the servings could have been fruit juice, which has been found to have no effect on fasting glucose and insulin [23]. It is possible that the pattern of eating the additional fruit may be relevant to glycaemia, as the postprandial glucose and insulin area under the curve were lower when two kiwifruit were eaten as a breakfast preload compared with a control breakfast without preload [13] However, consistent with other work, adding kiwifruit to the diet was not found to affect the fasting glucose concentration or blood lipids [24].

The finding of a difference in systolic blood pressure between groups is consistent with studies in which people have been randomised to eat more fruit or vegetables. Over a 6-month period, an increase of 1.4 (1.7) portions of fruit or vegetables resulted in a difference in the change from baseline of 4.0 (95% CI: 2.0, 6.0) mmHg systolic and 1.5 (0.2, 2.7) mmHg diastolic blood pressure between the control and intervention group [25]. Our between-group difference in systolic blood pressure of 2.7 mmHg was less than that found by John and colleagues [25]. Given that the increase in portions was similar between studies, the difference in the change in blood pressure could be due to study duration or the types of fruits and vegetables being eaten. However, our data are consistent with an 8-week study carried out by the Dietary Approaches to Stop Hypertension (DASH) Collaborative Research Group, in which participants following a diet rich in fruits and vegetables had systolic and diastolic blood pressure reductions of 2.8 and 1.1 mmHg, respectively, compared with the control group [16]. In that study, greater systolic and diastolic reductions of 5.5 and 3.0 mmHg, respectively, were found with a ‘combination’ diet that included high fruit/vegetable and low-fat dairy product intake, with the authors predicting clinically meaningful reductions in incident coronary heart disease and stroke with blood pressure reductions of this magnitude [16]. Our reduction in systolic blood pressure was approximately half of that attained with the combination diet used by the DASH Collaborative Research Group [16]; nevertheless, the consistency in direction among intervention studies suggests a beneficial effect on blood pressure by increasing the fruit content in a diet [24].

Fructose intake at breakfast increased in our kiwifruit intervention group by 4.4 g per day. This is less than the amount of fructose contained in two kiwifruit, analysed as 5.8 g per fruit [26]. The explanation is that the kiwifruit partially replaced other breakfast fruits and fruit juice, as evidenced by an increase of only 1.3 portions rather than 2 portions of fruit per day in the intervention group compared with the control group. Fructose added to hypercaloric diets in quantities equivalent to 25% of energy content has been found to have deleterious effects on markers of health [6]. However, in our study, there were no statistically significant effects on fasting uric acid, triglycerides, or total cholesterol following 7 weeks of kiwifruit consumption providing a small increment in fructose to the diet. Additionally, low-dose fructose infusion at a rate delivering approximately 11 g of fructose over 4 h stimulated net hepatic glycogen synthesis compared with a glucose control condition, with the authors suggesting a therapeutic benefit of low-dose fructose [27]. Thus, it seems unlikely that the small amount of fructose contained in two kiwifruit will cause a metabolic disturbance, and it may actually assist in hepatic glycogen storage. Additionally, kiwifruit have a low energy density with a healthful nutrient profile, being a good source of fibre, carotenoids, and vitamins C and E [26]. The results suggest that comorbidities associated with metabolic syndrome would not be worsened by the ingestion of two kiwifruit per day. Instead, because of the high intake of vitamin C that would result, there could be a range of benefits associated with vitamin C adequacy, including a reduction in blood pressure, as noted in a meta-analysis of blood pressure effects of vitamin C supplementation [28]. However, similarly, the blood pressure-lowering effects of increased potassium intake, noted in another recent meta-analysis, could contribute to the kiwifruit’s effects [29]. The exact mechanisms underlying the effects of vitamin C and potassium on blood pressure are uncertain, and they are not necessarily coupled to all of the parameters measured in the present study.

The experimental daily allocation of fruit (200 g) in the present study was associated with a systolic blood pressure reduction of 2.7 mmHg. For future research, a dose-response study may be useful, as an intake of 360 g kiwifruit per day was associated with a systolic blood pressure reduction of 3.7 mmHg over 8 weeks compared with the control group [30].

Although the amount of fibre in kiwifruit is comparable, on a dry weight basis, to other commonly consumed fruits, kiwifruit fibre has better swelling and water retention properties compared with apples and oranges [31]. Whether these kiwifruit fibre properties translate to better gastrointestinal functionality in humans compared with other fruit fibres remains untested, but in rats, stool weight was strongly dose-dependent on the kiwifruit content of the diet [31]. In humans, kiwifruit intervention trials have resulted in improvements in bowel movements [32,33].

It has been found that eating fruit and vegetables increases plasma antioxidant concentrations [25]. The antioxidant capacity of kiwifruit is comparable to that of grapefruit and oranges and exceeds that of other commonly consumed fruits, including apples, pears, and bananas [34]. It is possible, therefore, that the antioxidant capacity of carotenoids and vitamins C and E in kiwifruit offers a healthful combination of nutrients that may be of therapeutic benefit. In kiwifruit intervention trials in humans, measurable effects in reducing DNA oxidative damage and enhancing DNA repair have been found [35,36]. There is even the suggestion that additional vitamin C intake could alleviate oxidative stress caused by COVID-19 [37].

A strength of this study is the practical applicability of a kiwifruit intervention, with participants willing to incorporate the fruit into their breakfasts without increasing their energy intake, either at breakfast or throughout the day. Incorporating additional fruit into breakfast should help people meet daily fruit and vegetable recommendations. Another strength was the use of carbonated water as a means of providing motivational equality for people randomly allocated to the control and kiwifruit groups. It is important to control for preference in randomised controlled trials to avoid potential feelings of disappointment from being assigned to a control group [38].

A limitation to attributing group differences in outcome measures at 7 weeks solely to the kiwifruit intervention was the lower vegetable intake by the kiwifruit group at baseline compared with the control group, although vegetable intakes were similar between groups by 7 weeks. Another limitation is that generalisability is restricted due to the sample being predominantly female young adults of Asian descent. New Zealand has a multicultural population, and it may be of benefit to repeat this intervention with other ethnic groups with good male representation. At a population level, around half of males and one-third of females do not meet New Zealand’s recommended two servings of fruit per day [39]. Although kiwifruit has a good nutrient profile [26] and was acceptable to participants during the study period as a means to increase fruit consumption, a limitation to year-long consumption of kiwifruit in New Zealand is the short season of unavailability. There were also some limitations associated with the availability of resources, time available to participants and researchers, and the storage life of kiwifruit, which did not favour a long-term comprehensive study. Thus, the inclusion of a parallel positive control group consuming only the sugar components of kiwifruit may have indicated the specific role of sugars in any effects noted. A more in-depth analysis of plasma lipids such as HDL-cholesterol could have been conducted. A small increase in HDL-cholesterol and no effect on LDL-cholesterol was reported for men with hypercholesterolemia who consumed two kiwifruit per day. [40] Although a small number of studies related to the metabolic effects of kiwifruit have been conducted, they have generally used participants with already-established metabolic disorders or related conditions [24], unlike those in the present study, who were Asian and healthy at the start of the kiwifruit intervention.

## 5. Conclusions

In summary, we found that adding a small amount of kiwifruit to breakfast has potential health benefits without any evidence to suggest that a small increment in fructose intake causes a metabolic disturbance. Given the potential for a difference in fasting insulin and blood pressure between the control and intervention groups, in future research, it may be useful to recruit a larger sample and to continue the intervention for longer than 7 weeks.

## Figures and Tables

**Table 1:** Demographic characteristics by randomised group (n = 43).

Characteristic		Total	Control Group	Intervention Group
Sex	Male	11	7	4
	Female	32	16	16
Age, y—mean (SD)		21.9 (2.8)	21.9 (2.0)	21.9 (3.5)
BMI, kg/m^2^—mean (SD)		22.0 (3.0)	21.6 (3.4)	22.4 (2.5)

BMI = body mass index (n = 42); SD = standard deviation.

**Table 2:** Nutrient intake from breakfast before and during intervention (n = 32).

	Carbonated Water (n = 18)		Kiwifruit (n = 14)		Mean Difference (95% CI)	
	Baseline	Change at 4 wk ^a^	Baseline	Change at 4 wk ^a^	Change over 4 wk ^b^	*p*-Value
Energy (kJ)	1863 (706)	−277 (1176)	2100 (968)	−528 (774)	−398 (−1048, 251)	0.220
Fructose (g)	3.3 (3.1)	−1.0 (4.4)	7.1 (9.5)	4.4 (5.6)	7.6 (5.1, 10.2)	<0.001
Fibre (g)	5.8 (3.5)	−0.5 (4.7)	6.8 (5.0)	−1.7 (3.8)	−0.5 (−2.9, 1.8)	0.654
Vitamin C (mg)	11.9 (17.0)	−1.2 (32.4)	21.5 (47.2)	233.5 (80)	240 (198, 281)	<0.001
Vitamin E (mg)	3.0 (4.0)	−1.5 (4.3)	2.3 (1.6)	1.1 (2.0)	1.8 (0.9, 2.8)	0.001
Carbohydrate (% energy)	43.9 (16.6)	−1.1 (15.3)	56.0 (12.8)	8.0 (18.8)	16.0 (4.3, 27.7)	0.009
Fat (% energy)	37.7 (15.5)	−0.9 (14.9)	28.6 (10.7)	−6.6 (16.8)	−12.6 (−21.9, −3.2)	0.010
Protein (% energy)	17.4 (7.1)	2.4 (10.9)	14.3 (4.3)	−2.0 (6.7)	−3.2 (−10.1, 3.8)	0.355

^a^ Mean (SD) within-group change over 4 weeks; ^b^ mean differences, 95% CI, and *p*-values for the mean difference between the groups estimated using linear regression models adjusted for baseline. The adjustment for baseline means that the mean differences might not be equal to the difference in the mean changes, but they more accurately estimate the effects of the intervention.

**Table 3:** Effect of daily consumption of kiwifruit compared with carbonated water on metabolic outcomes (n = 42).

	Carbonated Water (n = 22)		Kiwifruit (n = 20)		Mean Difference (95% CI)	
	Baseline ^a^	Change at 7 wk ^a^	Baseline ^a^	Change at 7 wk ^a^	Change over 7 wk ^b^	*p*-Value
**Overnight fasting venous measures**						
Insulin (μU/mL)	8.5 (3.6)	2.1 (4.1)	8.7 (2.9)	0.3 (2.0)	−1.62 (−3.48, 0.25)	0.089
Glucose (mmol/L)	5.0 (0.3)	0.0 (0.3)	4.9 (0.4)	0.1 (0.3)	0.00 (−0.15, 0.16)	0.979
Uric acid (μmol/L)	304 (80)	9 (33)	288 (60)	−1 (26)	−13.0 (−30.4, 4.4)	0.144
Triglycerides (mmol/L)	0.87 (0.24)	0.06 (0.23)	0.88 (0.44)	−0.05 (0.31)	−0.10 (−0.22, 0.03)	0.126
Total cholesterol (mmol/L)	4.5 (1.1)	0.0 (0.4)	4.4 (0.8)	0.0 (0.3)	−0.05 (−0.24, 0.14)	0.584
Oral glucose tolerance test ^c^ iAUC (mmol/L × min)	273 (135)	4 (140)	262 (120)	55 (81)	46 (−19, 111)	0.163
**Overnight fasting capillary measures**						
Glucose (mmol/L)	5.1 (0.4)	0.0 (0.3)	5.0 (0.5)	0.1 (0.4)	0.09 (−0.06, 0.24)	0.224
**Blood pressure**						
Systolic (mmHg)	112 (8)	1 (6)	111 (11)	−1 (4)	−2.7 (−5.5, 0.0)	0.048
Diastolic (mmHg)	65 (5)	−1 (5)	68 (7.5)	−4 (6)	−1.4 (−4.4, 1.6)	0.368
**Anthropometry**						
Weight (kg)	60.4 (9.3)	0.1 (2.3)	59.0 (13.1)	0.1 (1.1)	−0.12 (1.19, 0.94)	0.822
BMI (kg/m^2^)	22.4 (2.5)	0.1 (0.9)	21.6 (3.3)	0.0 (0.4)	−0.12 (−0.60, 0.32)	0.591
Waist circumference (cm)	73 (7)	0 (3)	74 (9)	0 (2)	0.3 (−1.1, 1.8)	0.644

^a^ Mean (SD) baseline values are the average from visits 1 and 2; mean (SD) change at 7 wk is the change from baseline to the average of visits 5 and 6. ^b^ Mean differences, 95% CI, and *p*-values for the mean difference between the groups in change of outcome estimated using mixed-effect regression with participant as a random effect. The adjustment for baseline means that the mean differences might not be equal to the difference in the mean changes, but they more accurately estimate the effects of the intervention. ^c^ Oral glucose tolerance test incremental area under the curve (iAUC); one participant in the kiwifruit group was missing data.

## Data Availability

For data please contact the corresponding author.
